# Ectopic clustering of Cajal–Retzius and subplate cells is an initial pathological feature in *Pomgnt2*-knockout mice, a model of dystroglycanopathy

**DOI:** 10.1038/srep11163

**Published:** 2015-06-10

**Authors:** Naoki Nakagawa, Hirokazu Yagi, Koichi Kato, Hiromu Takematsu, Shogo Oka

**Affiliations:** 1 Department of Biological Chemistry, Human Health Sciences, Graduate School of Medicine, Kyoto University, 53 Kawahara-cho, Shogoin, Sakyo-ku, Kyoto 606-8507, Japan; 2 Graduate School of Pharmaceutical Sciences, Nagoya City University, 3-1 Tanabe-dori, Mizuho-ku, Nagoya 467-8603, Japan; 3 Okazaki Institute for Integrative Bioscience and Institute for Molecular Science, National Institutes of Natural Sciences, 5-1 Higashiyama Myodaiji, Okazaki 444-8787, Japan

## Abstract

Aberrant glycosylation of dystroglycan causes congenital muscular dystrophies associated with cobblestone lissencephaly, classified as dystroglycanopathy. However, pathological features in the onset of brain malformations, including the precise timing and primary cause of the pial basement membrane disruption and abnormalities in the migration of pyramidal neurons, remain unexplored. Using the *Pomgnt2*-knockout (KO) mouse as a dystroglycanopathy model, we show that breaches of the pial basement membrane appeared at embryonic day 11.5, coinciding with the ectopic clustering of Cajal–Retzius cells and subplate neurons and prior to the migration onset of pyramidal neurons. Furthermore, in the *Pomgnt2*-KO cerebral cortex, preplate splitting failure likely occurred due to the aggregation of Cajal–Retzius and subplate cells, and migrating pyramidal neurons lost polarity and radial orientation. Our findings demonstrate the initial pathological events in dystroglycanopathy mice and contribute to our understanding of how dystroglycan dysfunction affects brain development and progresses to cobblestone lissencephaly.

Dystroglycanopathy encompasses a group of congenital muscular dystrophies (CMDs), including Walker–Warburg syndrome, muscle–eye–brain disease, Fukuyama CMD, and several forms of limb-girdle muscular dystrophies^1^. Dystroglycanopathy shows highly heterogeneous clinical symptoms, ranging from CMDs with disorganized brain architecture, mental retardation, and eye abnormalities in the most severe cases to adult-onset limb-girdle muscular dystrophy without brain and eye malformations in milder cases^1^. The common molecular feature found in dystroglycanopathy is a deficiency in the glycosylation of dystroglycan, which diminishes the ligand-binding activity of this glycoprotein^1^,^2^. Dystroglycan is a cell surface receptor for several extracellular matrix (ECM) proteins, such as laminin, agrin, and perlecan, and plays a central role in the generation of physical and functional linkages between the cell and the ECM^3^,^4^. Dystroglycan is ubiquitously expressed in a wide variety of organs^4^, which leads to abnormalities in multiple tissues when its function is compromised as in dystroglycanopathy. Although the pathogenesis of muscle defects has been extensively studied^5^,^6^,^7^, brain defects are not fully characterized, especially with regard to neurodevelopmental alteration in the initial phase of brain malformation.

The ligand-binding glycan expressed on dystroglycan is a unique phosphodiester-linked polysaccharide structure built on the *O*-mannose attached to the dystroglycan core protein, which is known as the so-called post-phosphoryl modification^8^. In the glycosylation pathway of dystroglycan, the phosphorylated trisaccharide [GalNAcβ1-3GlcNAcβ1-4(phosphate-6)Man] is first generated on dystroglycan and then the linear heteropolymer with ligand-binding ability, which is composed of repeated units of (–3Xylα1-3GlcAβ1–), is elongated from the trisaccharide presumably via a phosphodiester linkage^9^,^10^. However, the linking structure connecting the ligand-binding heteropolymer to the phosphorylated trisaccharide is currently unknown. Many genes encoding known or putative glycosyltransferases are mutated in patients with dystroglycanopathy^11^. Recent studies have demonstrated that some, but not all, of those gene products actually possess glycosyltransferase activity, which is directly or indirectly required for the assembly of the ligand-binding glycan on dystroglycan^9^,^10^,^12^,^13^,^14^,^15^,^16^,^17^,^18^. For example, the attachment of a mannose to the core protein is catalyzed by an enzyme complex of protein *O*-mannosyltransferase 1 (POMT1) and POMT2^16^, and the terminal heteropolymer is synthesized by like-acetylglucosaminyltransferase (LARGE)^9^. Moreover, we and other researchers recently identified protein *O*-mannose β-1,4-*N*-acetylglucosaminyltransferase 2 (POMGNT2) [also known as glycosyltransferase-like domain-containing 2 (GTDC2) or AGO61] as a β-1,4-*N*-acetylglucosaminyltransferase that synthesizes the GlcNAcβ1-4Man structure in the phosphorylated trisaccharide^10^,^17^,^18^.

Structural brain abnormality in dystroglycanopathy is a type of neuronal migration disorder classified as a type II lissencephaly, also known as cobblestone lissencephaly^19^. In dystroglycanopathy, the pial basement membrane is disrupted due to the lack of dystroglycan function, and neurons over-migrate beyond the breached basement membrane, resulting in the formation of a neuronal heterotopia and perturbation of the six-layered structure of the neocortex^19^. Disorganized morphologies of radial glial fibers are also observed. Accumulating evidence indicates that, in the developing brain, dystroglycan is specifically located at the radial glial end feet and plays a critical role in the maintenance of pial basement membrane integrity^20^,^21^. In contrast, dystroglycan is absent in immature postmitotic neurons during their migration phases, whereas it appears in differentiating neurons along with dendritic maturation and co-localizes with GABA_A_ receptors at inhibitory synapses^21^,^22^. Furthermore, the neuron-specific depletion of dystroglycan in mice causes no overt pathological changes in brain architecture^23^, indicating that the neuronal migration defect may be caused by environmental changes, and not by an alteration of the neuron itself. These findings reveal the final outcome of brain defects in dystroglycanopathy and suggest that the abnormalities are derived as a secondary defect of neuronal migration due to the dysfunction of radial glial cells. However, the initial pathological changes in the neocortical environment and their effects on migrating neurons, leading to over-migration, remain unclear.

To clarify the pathogenesis of dystroglycanopathy-associated brain malformation, we analyzed the *Pomgnt2*-knockout (KO) mouse brain as a disease model by focusing on its early developmental stages. We observed that Cajal–Retzius cells and subplate neurons formed ectopic clusters in the meningeal spaces in the *Pomgnt2*-KO brain as early as embryonic day 11.5 (E11.5). The ectopic cell cluster formation coincided with the disruption of the pial basement membrane, but preceded the over-migration of excitatory neurons. Moreover, the polarity and radial orientation of migrating excitatory neurons were disrupted in the *Pomgnt2*-KO cortex. Our findings contribute to the understanding of initial pathological events in the developing brain of the dystroglycanopathy mouse model, which lead to cobblestone lissencephaly.

## Results

### Basement membrane disruption and ectopic cell clusters are obvious at E12.5 in the *Pomgnt2*-KO brain

**Figure 1:**
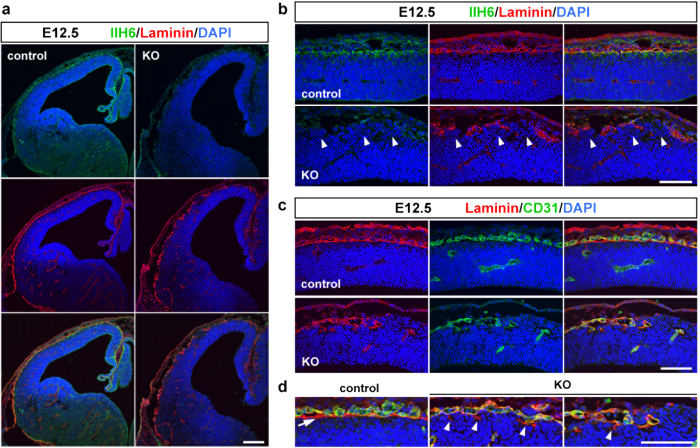
The Pomgnt2-KO cerebral cortex at E12.5 shows severe defects in pial basement membrane integrity. (**a**) Coronal sections of the developing brain from control and *Pomgnt2*-KO embryos at E12.5 were immunostained with IIH6 mAb and anti-laminin pAb. (**b**) Enlarged images of the dorsal cortex in (**a**). Arrowheads indicate ectopically located cells at gaps of discontinuous laminin signals. (**c**) Immunohistochemistry for laminin and CD31 at E12.5. (**d**) Enlarged images of the pial surface in (**c**). The arrow indicates the laminin^+^/CD31^−^ pial basement membrane in the control cortex and arrowheads show fragmented structures derived from the basement membrane in the *Pomgnt2*-KO cortex. Scale bars represent 200 μm (**a**) and 100 μm (**b**–**d**).

The *Pomgnt2*-KO mouse shows a lack of the laminin-binding activity of dystroglycan^18^. Thus, while the loss of POMGNT2 causes perinatal death, *Pomgnt2*-KO mice could be useful for analyzing the development of the cerebral cortex until birth as a dystroglycanopathy model. It remains unclear whether the pial basement membrane disruption in dystroglycanopathy is caused by migrating pyramidal neurons or other pathological events. Therefore, determining whether the pial basement membrane disruption is coincident with or already in progress at the radial migration phase of pyramidal neurons is important. In the mouse cerebral cortex, the first population of pyramidal neurons (future layer VI neurons) is formed at around E12.5 and subsequently migrates toward the pial surface^24^,^25^. To investigate the neurodevelopmental alterations in the *Pomgnt2*-KO mouse at E12.5, a coronal section of the cerebral cortex was analyzed using immunohistochemistry. Since the phenotype of the *Pomgnt2* heterozygous embryo is grossly normal and indistinguishable from the wild type^18^, the wild type and heterozygous brains were used as controls in this study. In the control brain, the immunoreactivity of IIH6, a monoclonal antibody that recognizes the ligand-binding glycan on dystroglycan, was observed at the pial surface in association with the basement membrane visualized by laminin immunostaining (Fig. 1a,b). This result indicated that dystroglycan localizes at the end feet of radial glial cells, consistent with a previous study^21^. In contrast, IIH6 immunoreactivity was absent in the *Pomgnt2*-KO brain (Fig. 1a,b), which confirmed that POMGNT2 is required for the glycosylation of dystroglycan. Moreover, in the *Pomgnt2*-KO mouse, the laminin signal at the pial surface was discontinuous and ectopic cell clusters were present (Fig. 1a,b).

Laminin is present at both the pial basement membrane and meningeal blood vessel wall in the brain. Thus, immunostaining for CD31, a vascular endothelial cell marker, was performed to distinguish these two structures. In the control brain at E12.5, a single layer of the laminin^+^/CD31^–^ pial basement membrane was visible just beneath the laminin^+^/CD31^+^meningeal blood vasculature (Fig. 1c,d). However, in the *Pomgnt2*-KO cortex, co-labeling for laminin and CD31 revealed that most laminin signals at the pial surface were located in the blood vessel, and also highlighted that fragmentary remnants of the pial basement membrane were present (Fig. 1c,d). These results demonstrated that the pial basement membrane had been severely disrupted before the arrival of migrating pyramidal neurons.

### The pial basement membrane is formed at E10.5 but disrupted at E11.5 in the *Pomgnt2*-KO brain

**Figure 2:**
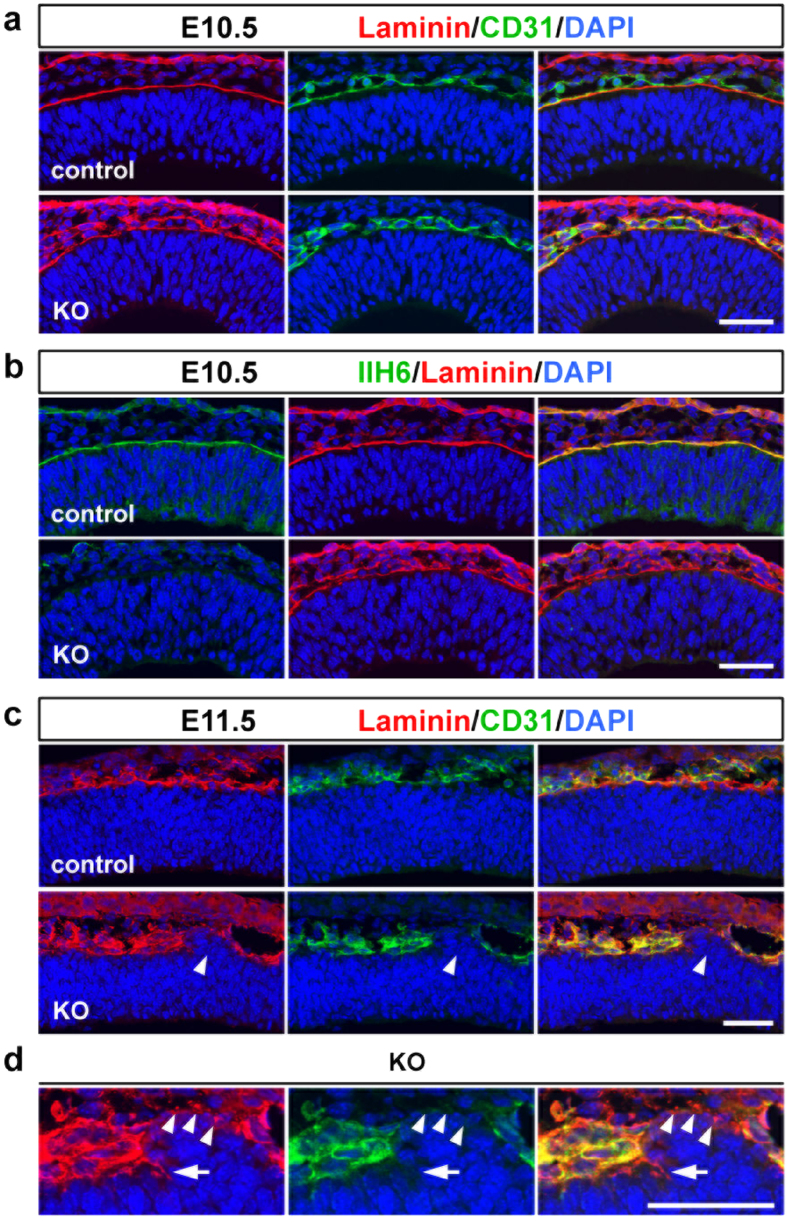
The pial basement membrane in the Pomgnt2-KO cortex begins to be disrupted at E11.5. (**a** and **b**) Coronal sections of the forebrain from the control and *Pomgnt2*-KO embryos at E10.5 were immunostained with anti-laminin pAb and anti-CD31 pAb (**a**) and IIH6 mAb and anti-laminin pAb (**b**). (**c**) Coronal sections of the cerebral cortex from control and *Pomgnt2*-KO embryos at E11.5 were immunostained for laminin and CD31. Arrowheads indicate the emergence of the ectopic cell cluster at the gap in the breach of the pial basement membrane visualized by laminin immunostaining in the *Pomgnt2*-KO brain. (**d**) Enlarged images of the pial surface of the E11.5 *Pomgnt2*-KO brain in (**c**). Arrowheads indicate that laminin^+^/CD31^–^ fragments derived from the pial basement membrane were observed over the ectopic cell cluster. The arrow points to the gap in the pial basement membrane. Scale bars represent 50 μm.

To determine the onset of basement membrane disruption, we analyzed the E10.5 *Pomgnt2*-KO forebrain. In the control forebrain, a single line of pial basement membrane was detected by co-immunostaining for laminin and CD31 (Fig. 2a). IIH6-immunoreactivity was also found in association with the pial basement membrane in the control cortex, which disappeared in the *Pomgnt2*-KO cortex (Fig. 2b), demonstrating that dystroglycan was already expressed at E10.5 and that its ligand-binding glycans were absent in the *Pomgnt2*-KO brain. Despite the loss of functional dystroglycan, a continuous basement membrane was present in the *Pomgnt2*-KO brain at E10.5 (Fig. 2a), which suggests that the dystroglycan dysfunction does not affect the construction of the pial basement membrane. Next, we examined the E11.5 *Pomgnt2*-KO brain and found breaches of the pial basement membrane at this stage (Fig. 2c). Moreover, an ectopic cell cluster was emerging at the gap in the disrupted basement membrane (Fig. 2c). Tiny fragments apparently derived from the basement membrane were observed over the ectopic cell cluster (Fig. 2d), which implies that the aberrant localization of these cells may trigger basement membrane breakdown. Taken together, these results indicate that the ligand-binding glycan-dependent function of dystroglycan is not crucial for the assembly of the basement membrane, but is essential for its maintenance.

### Cajal–Retzius cells and subplate neurons are abnormally distributed and form ectopic clusters

**Figure 3:**
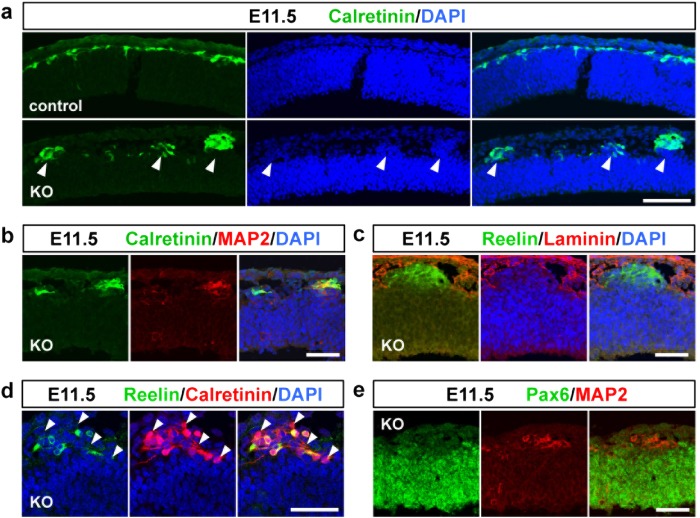
Abnormally located Cajal–Retzius cells and subplate neurons constitute ectopic clusters. (**a**) Coronal sections of the developing brain from control and *Pomgnt2*-KO embryos at E11.5 were immunostained using anti-calretinin pAb. Arrowheads indicate that ectopic cell clusters present in the E11.5 *Pomgnt2*-KO cortex were composed of calretinin^+^cells. (**b–e**) Immunohistochemistry of the E11.5 *Pomgnt2*-KO cortex for calretinin and MAP2 (**b**), reelin and laminin (**c**), reelin and calretinin (**d**), and Pax6 and MAP2 (**e**). Arrowheads in (**d**) indicate the presence of calretinin^+^/reelin^−^ cells in the ectopic cluster. Note that the MAP2^+^cell cluster was negative for Pax6 (**e**). Scale bars represent 100 μm (**a**) and 50 μm (**b–e**).

Next, we attempted to identify what kinds of cells populated the ectopic cluster observed in the *Pomgnt2*-KO cortex at E11.5. Three major cell types exist in the E11.5 dorsal cortex: Cajal–Retzius cells, subplate neurons, and radial glial cells^24^,^25^. Therefore, immunostaining for their marker proteins was performed. Calretinin^+^cells comprising Cajal–Retzius cells and subplate neurons formed a single layer just beneath the pia mater in the control cortex, but aggregated at the ectopic clusters in the *Pomgnt2*-KO cortex (Fig. 3a). The ectopic cell clusters were also positive for MAP2 and reelin (Fig. 3b,c), and contained calretinin^+^/reelin^+^cells as well as calretinin^+^/reelin^−^ cells (Fig. 3d), indicating the presence of both Cajal–Retzius cells and subplate neurons in the heterotopia. Pax6^+^radial glial cells were not observed in the ectopic cluster (Fig. 3e).

**Figure 4:**
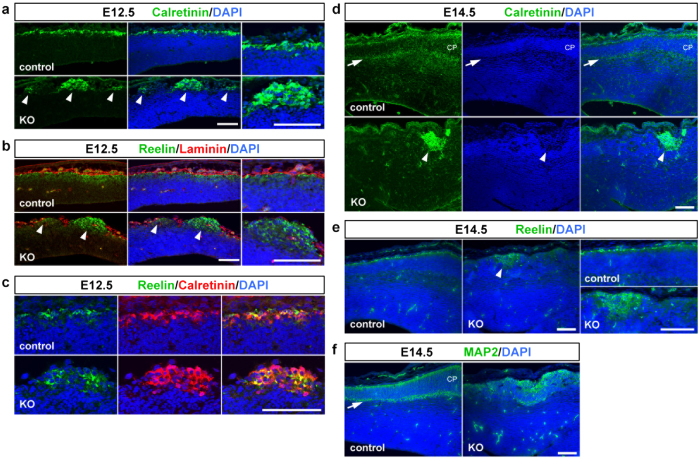
The calretinin-positive heterotopia is retained along with the development of the Pomgnt2-KO brain. (**a–c**) Coronal sections of the developing brain from control and *Pomgnt2*-KO embryos at E12.5 were immunostained for calretinin (**a**), reelin and laminin (**b**), and reelin and calretinin (**c**). Arrowheads in (**a**) and (**b**) indicate that the ectopic cell clusters observed in the E12.5 *Pomgnt2*-KO cortex contained calretinin^+^and reelin^+^cells, respectively. Far right panels in (**a**) and (**b**) are the magnified images of the pial surface showing that calretinin^+^(**a**) and reelin^+^(**b**) cells formed a single layer in the control cortex, but aggregated in the *Pomgnt2*-KO cortex. Note that both calretinin^+^/reelin^+^and calretinin^+^/reelin^−^ cells were present at the ectopic cluster (**c**). (**d** and **e**) Immunohistochemistry for calretinin (**d**) and reelin (**e**) at E14.5. Arrowheads in (**d**) and (**e**) indicate that calretinin^+^and reelin^+^ectopic cell clusters, respectively, were still present in the E14.5 *Pomgnt2*-KO cortex. The calretinin^+^layer shown by the arrow in (**d**) is the subplate, indicating that the cortical plate (CP) was formed in the control cortex. Far right panels in (**e**) are the magnified images of the pial surface. (**f**) Immunohistochemistry for MAP2 at E14.5. The arrow indicates the subplate formed in the control cortex. Scale bars represent 100 μm.

We assessed the alteration of the ectopic cluster along with cortical development. Ectopic clusters composed of Cajal–Retzius cells and subplate neurons, which were formed at E11.5, were enlarged in size at E12.5 and still observed at E14.5 in *Pomgnt2*-KO brains (Fig. 4a–e). In the E14.5 control brain, substantial amounts of pyramidal neurons had migrated to the upper region of the neocortex and formed the cortical plate (CP). Calretinin immunostaining revealed the splitting of the preplate and the appearance of the subplate (Fig. 4d), a boundary layer between the CP and intermediate zone composed of calretinin^+^subplate neurons^26^. In contrast, the subplate was not observed in the *Pomgnt2*-KO brain at E14.5 (Fig. 4d). MAP2 immunostaining also revealed the disruption of the subplate formation in the *Pomgnt2*-KO cortex (Fig. 4f). These data indicate the failure of the preplate splitting in the *Pomgnt2*-KO brain, presumably due to the ectopic aggregation of Cajal–Retzius cells and subplate neurons that would normally be split by climbing pyramidal neurons.

### The polarized morphology and radial orientation of excitatory neurons are disrupted in the *Pomgnt2*-KO cortex

**Figure 5:**
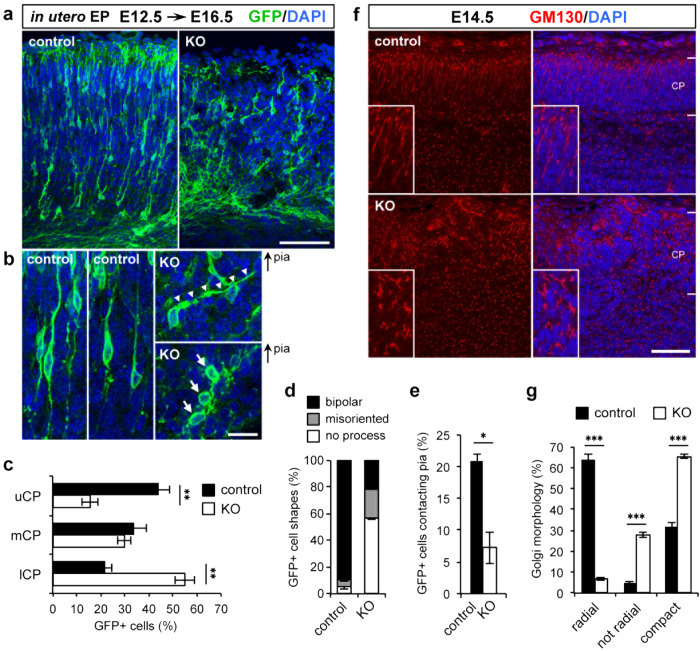
Excitatory neurons in the Pomgnt2-KO cortex display aberrant migratory behaviors. (**a**) Coronal sections of E16.5 control and *Pomgnt2*-KO brains following *in utero* electroporation with the pCAGGS-GAP-Venus plasmid (encoding a modified GFP variant) at E12.5. Immunostaining for GFP visualizes the distribution and morphology of the migrating neurons. (**b**) Enlarged images showing a closer view of the migrating neurons in (**a**). In the *Pomgnt2*-KO cortex, neurons with an incorrectly oriented leading process (indicated by arrowheads) or with no leading process (indicated by arrows) were observed. Panels are placed with the pial surface to the top. (**c–e**) Quantification of the distribution of GFP^+^cells in different zones of the CP (**c**), morphology of GFP^+^cells (**d**), and percentage of GFP^+^cells contacting with the pial surface (**e**). uCP, upper CP; mCP, median CP; lCP, lower CP. The graph shows the mean ± SEM from 3 embryos for each genotype. Student’s *t*-test; *P < 0.05, **P < 0.01. (**f**) Coronal sections of the developing brain from control and *Pomgnt2*-KO embryos at E14.5 were immunostained for GM130 to analyze the morphology of the Golgi apparatus. Magnified images of the Golgi apparatus in the CP are shown in the insets. (**g**) Golgi morphology was assigned to three categories (radial, not radial, and compact), and the number of Golgi bodies in each category was quantified. The graph shows the mean ± SEM from 4 embryos for each genotype. Student’s *t*-test; ***P < 0.001. Scale bars represent 100 μm (**a**), 20 μm (**b**), and 50 μm (**f**).

Since the highly ordered spatial arrangements of Cajal–Retzius cells and subplate neurons are essential for the proper guidance of the radial migration of pyramidal neurons^24^,^25^, our observation of ectopic cluster formation and preplate splitting failure suggests that the migration of pyramidal neurons is disorganized in the *Pomgnt2*-KO brain. Therefore, we investigated how these events in the early developmental stages affected the migration of excitatory neurons, which could lead to over-migration and the cobblestone phenotype. The morphology of radially migrating neurons in the *Pomgnt2*-KO cortex was examined by labeling the newborn neurons with green fluorescent protein (GFP) using *in utero* electroporation. A plasmid expressing GFP was electroporated into progenitor cells in the ventricular zone of the dorsal cortex at E12.5 to visualize early-born excitatory neurons. We then analyzed the positions and morphologies of neurons generated from these progenitor cells at E16.5. In the control brain, a substantial number of neurons (44.1 ± 4.4%) reached the upper region of the CP at E16.5 (Fig. 5a,c). In contrast, many neurons did not enter the upper CP and remained in the lower CP (upper CP, 15.5 ± 3.2%; lower CP, 54.9 ± 4.2%) in the *Pomgnt2*-KO brain at E16.5 (Fig. 5a,c). Moreover, although most of the migrating neurons in the control brain exhibited the bipolar morphology with a leading process oriented toward the pial surface, the number of neurons with typical bipolar morphology was remarkably diminished in the *Pomgnt2*-KO brain (control, 89.7 ± 2.6%; *Pomgnt2*-KO, 21.3 ± 0.5%) (Fig. 5a,b,d). Furthermore, many neurons had a misoriented (control, 4.9 ± 0.9%; *Pomgnt2*-KO, 22.1 ± 0.6%) or no (control, 5.3 ± 1.7%; *Pomgnt2*-KO, 56.6 ± 0.8%) leading process (Fig. 5a,b,d), indicating that the polarity of migrating neurons is lost in the *Pomgnt2*-KO cortex.

The polarity of excitatory neurons during radial migration was further evaluated by the morphology of the Golgi apparatus. In excitatory neurons during the radial migration phase, the Golgi apparatus is localized ahead of the nucleus and extends toward the pial surface^27^,^28^. GM130 immunostaining revealed that a large number of neurons (“radial” in Figs. 5g, 63.9 ± 2.3%) had a typically elongated Golgi apparatus facing in the migratory direction in the control CP (Fig. 5f,g). In contrast, in the *Pomgnt2*-KO CP, the number of Golgi with a radially oriented morphology was markedly reduced (“radial” in Figs. 5g, 6.8 ± 0.8%), while those with an extended but incorrectly oriented shape (“not radial” in Figs. 5g, 27.8 ± 1.2%) and globular and compact shapes (“compact” in Figs. 5g, 65.4 ± 0.9%) were increased (Fig. 5f,g). These results demonstrated the loss of polarity and radial orientation of migrating excitatory neurons in the *Pomgnt2*-KO brain. In the developing mouse brain, early-born neurons (destined for layers V and VI) and late-born neurons (destined for layers II to IV) differentially utilize the radial glia-independent and radial glia-guided modes of radial migration, respectively^29^,^30^. The radial glia-guided migration is thought to be disrupted in dystroglycanopathy because radial glial fibers show abnormal morphologies due to the lack of dystroglycan function^19^,^21^. In this regard, our findings obtained from analyses of early-born pyramidal neurons revealed that the radial glia-independent migration was also disorganized in dystroglycanopathy.

### Detachment of leading processes from the pial surface is a likely cause of neuronal migration defects in the *Pomgnt2*-KO brain

**Figure 6:**
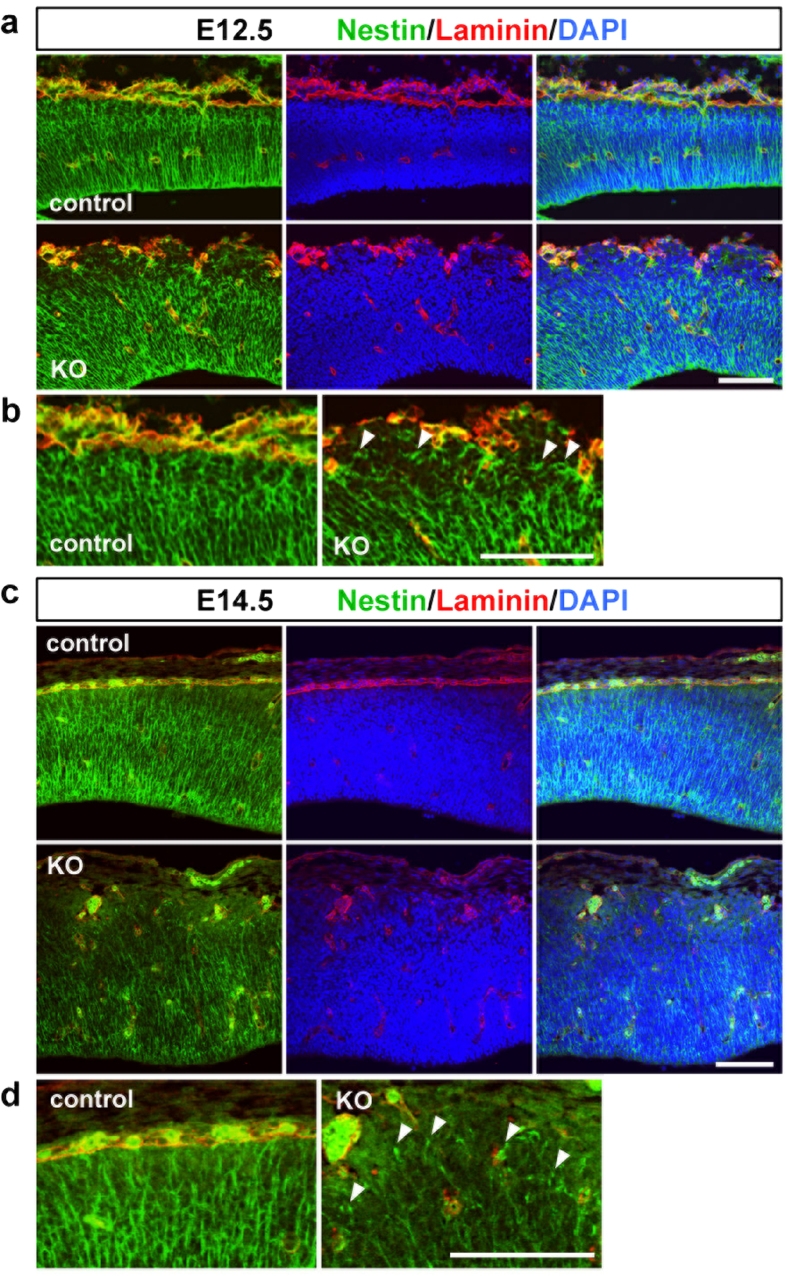
Nestin-positive fibers are detached from the pial surface in the Pomgnt2-KO cortex. (**a–d**) Coronal sections of the developing brain from control and *Pomgnt2*-KO embryos at E12.5 (**a** and **b**) and E14.5 (**c** and **d**) were immunostained for nestin and laminin. Magnified images of the pial surface in (**a**) and (**c**) are shown in (**b**) and (**d**), respectively. Arrowheads indicate the incorrectly terminated fibers that did not penetrate the entire thickness of the cerebral cortex, which were typically observed at aberrant cell aggregates. Scale bars represent 100 μm.

We investigated the pathological mechanism underlying the perturbation of the neuronal migration in the *Pomgnt2*-KO brain. The decrease in the number of neurons that enter the upper CP in the *Pomgnt2*-KO brain (Fig. 5a,c) is suggestive of a failure of somal translocation, the dynamic movement of neurons to reach their final destinations^29^,^30^. During somal translocation, neurons extend and attach their leading processes to the pial surface and then raise their cell bodies by utilizing their leading processes as anchorages^29^,^30^. Physical interactions between the neuronal leading process and ECM proteins in the marginal zone (MZ) or the Cajal–Retzius cell membrane are important for somal translocation^31^,^32^. In the *Pomgnt2*-KO cerebral cortex, the pial basement membrane is absent and the MZ structure is disorganized because of ectopic cluster formation (Figs 1, 2, 3). Therefore, neurons may not be able to maintain their leading processes due to the loss of anchorage points, and thus fail to raise their cell bodies. Consistent with this hypothesis, in the *Pomgnt2*-KO cortex, neurons in contact with the pial surface were decreased compared to those in the control cortex (control, 21.0 ± 1.0%; *Pomgnt2*-KO, 7.2 ± 2.5%) (Fig. 5a,e). Moreover, in the *Pomgnt2*-KO brain at E12.5 and E14.5, nestin-positive fibers appeared to be sparse at the pial surface and terminated without reaching the top of the cortical wall, indicating that the basal processes of radial glial cells were detached and retracted from the pial surface, while these were in close contact with the pial basement membrane in the control brain (Fig. 6a–d). This observation also supports the above possibility because early-born excitatory neurons that migrate at around E14.5 have been shown to inherit nestin-positive basal processes from parental radial glial cells and use them as anchorages in somal translocation^33^,^34^.

**Figure 7:**
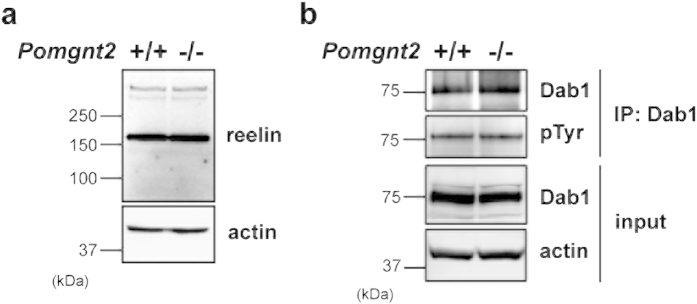
Reelin signaling is unchanged in the Pomgnt2-KO brain. (**a** and **b**) Brain lysates prepared from control and *Pomgnt2*-KO embryos at E16.5 were analyzed by immunoblotting. Actin was used as a loading control. Total amounts of reelin and Dab1 were assessed using anti-reelin mAb (**a**) and anti-Dab1 pAb (**b**, input), respectively. The phosphorylation level of Dab1 was evaluated by immunoblotting with anti-phosphotyrosine (pTyr) mAb using samples immunoprecipitated with the anti-Dab1 antibody (**b**, IP: Dab1). IP, immunoprecipitation. The full-length blots with anti-reelin, anti-Dab1, and anti-pTyr antibodies are presented in Supplementary Figs. S1a and S1b.

Accumulating evidence shows that reelin signaling is essential for neurons to undergo somal translocation^28^,^30^,^31^,^32^. Therefore, the mislocalization of Cajal–Retzius cells, which are major reelin-producing cells, may lead to alteration in the availability of reelin and/or the efficacy of reelin signaling, contributing to the defects in neuronal migration in the *Pomgnt2*-KO brain. To test this possibility, we analyzed the expression level of reelin and the phosphorylation level of disabled homolog 1 (Dab1), an intracellular adaptor protein that undergoes phosphorylation upon the binding of reelin to its receptors^35^. Immunoblotting analyses using lysates prepared from control and *Pomgnt2*-KO brains at E16.5 showed that the *Pomgnt2*-KO brain contained similar amounts of reelin as the control brain (Fig. 7a). Moreover, no obvious difference was observed in the phosphorylation level or in the total amount of Dab1 between the two genotypes (Fig. 7b). These results indicate that reelin signaling is not altered in the *Pomgnt2*-KO brain and is not responsible for the neuronal migration deficiency. Taken together, these findings suggest that the detachment of the leading process from the pial surface due to basement membrane disruption and ectopic cluster formation is the likely cause of neuronal migration defects in the *Pomgnt2*-KO brain.

### The laminar organization is severely disrupted in the *Pomgnt2*-KO cortex

**Figure 8:**
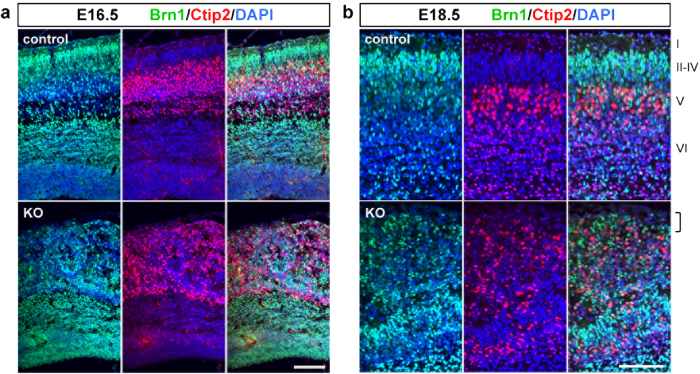
The Pomgnt2-KO cortex shows an abnormal cortical lamination. (**a** and **b**) Coronal sections of the developing brain from control and *Pomgnt2*-KO embryos at E16.5 (**a**) and E18.5 (**b**) were immunostained for brn1 (layers II-IV) and ctip2 (layer V). The bracket in (**b**) shows that the most superficial region of the cortex is occupied by over-migrated neurons in the *Pomgnt2*-KO brain, where the cell-sparse layer I is formed in the control brain. Scale bars represent 100 μm.

The pathological alterations observed in the developing brain of *Pomgnt2*-KO mice, including the preplate splitting failure and neuronal migration defects, raised a possibility that the laminar organization of the neocortex is disrupted. Therefore, we examined the cortical lamination in the *Pomgnt2*-KO brain using brn1 and ctip2 as upper- and deep-layer markers, respectively. In contrast to the control cortex where brn1^+^neurons settled superficially to ctip2^+^neurons, brn1^+^and ctip2^+^neurons were intermixed in the *Pomgnt2*-KO cortex at E16.5 and E18.5 (Fig. 8a,b). Moreover, whereas the most superficial region of the cerebral cortex was the cell-sparse layer (layer I) in the control brain at E18.5, the region was filled by over-migrated neurons in the *Pomgnt2*-KO brain (Fig. 8b). Both brn1^+^and ctip2^+^neurons were present at the heterotopia (Fig. 8b). These results indicate that both upper- and deep-layer projection neurons show severe migration defects in the *Pomgnt2*-KO brain, leading to the disruption of the cortical lamination in addition to neuronal over-migration.

## Discussion

The ligand-binding glycan on dystroglycan serves as a critical mediator for cell–ECM interaction and its disruption leads to dystroglycanopathy, a subset of CMDs affecting brain development and skeletal muscles^1^. The glycosylation-dependent function of dystroglycan is well established in skeletal muscle, where it acts as a member of the dystrophin–glycoprotein complex to link the muscle cell cytoskeleton with the surrounding ECM components^4^. The loss of functional dystroglycan increases the susceptibility of the sarcolemma to contraction-induced damage, resulting in muscle cell death and myofiber degeneration^5^,^6^,^7^. Dystroglycan is also important in sustaining the integrity of the pial basement membrane in the brain, and its dysfunction leads to neuronal over-migration and cobblestone lissencephaly^19^. The pathological features of the initiation phase of the structural brain abnormalities, such as the detailed timing and main cause of pial basement membrane disruption and behavioral changes in pyramidal neurons during radial migration, remain to be discovered. By analyzing the pathological alterations in the early developmental stages of the *Pomgnt2*-KO mouse brain, we provide deeper insights into how loss of dystroglycan function gives rise to the brain malformations in dystroglycanopathy.

By focusing on early developmental stages, we found that the pial basement membrane was breached at E11.5 in *Pomgnt2*-KO mice (Fig. 2c and d), coincident with the ectopic clustering of calretinin^+^cells (Fig. 3a). Several mouse models have been established to investigate the brain defects in dystroglycanopathy. Because the genetic ablation of dystroglycan (*Dag1*) in mice leads to early embryonic lethality at E7.5^36^, conditional KO (cKO) mice have been generated to yield the brain-specific deletion of dystroglycan and longer survival^20^,^23^. Glial fibrillary acidic protein (GFAP)-Cre- or Nestin-Cre-mediated deletion of dystroglycan in the central nervous system recapitulated the structural brain defects found in patients with dystroglycanopathy, such as basement membrane disruption, neuronal migration defects, and neuronal heterotopia formation^20^,^23^. Moreover, using these model mice, it has been shown that dystroglycan in radial glial cells, but not in neurons, is important in maintaining the brain architecture^23^, and that proper axonal projections to appropriate targets are observed despite the mispositioning of pyramidal neurons^21^. These model mice are useful for clarifying and characterizing the final outcome of brain abnormalities in dystroglycanopathy, but the analysis of disease onset is limited due to the residual effects of dystroglycan produced before Cre-mediated inactivation. Indeed, dystroglycan cKO (*Nestin-Cre*/*Dag1*^*lox/lox*^) mice exhibit a delayed onset of basement membrane disruption at E15.5^21^ compared to *Pomgnt2*-KO mice, in which dystroglycan function is disrupted throughout development (Fig. 2c,d). Mice lacking *O-mannose β-1,2-N-acetylglucosaminyltransferase 1* (*Pomgnt1*), one of the genes involved in dystroglycanopathy, also develop cobblestone lissencephaly-like cortical dysplasia^37^. As in *Pomgnt2*-KO mice, *Pomgnt1*-KO mice show defects in the glycosylation of dystroglycan throughout development, but these two mice differ in their disease onsets. The pial basement membrane is intact at E11.5 and rapidly disrupted by E13.5 in *Pomgnt1*-KO mice^37^. Of note, the precise role of POMGNT1 in production of the ligand-binding glycan on dystroglycan has not yet been clarified. POMGNT1 can affect the glycan composition of dystroglycan in an independent way from post-phosphoryl modification^13^, which may account for the apparent phenotypic divergence between *Pomgnt2*- and *Pomgnt1*-KO mice. Therefore, the *Pomgnt2*-KO mouse could be a useful model to investigate the early phase of dystroglycanopathy-associated brain malformation. For the future expansion of studies on dystroglycanopathy, utilizing the appropriate mouse model is important in accordance with the period when the pathological changes of interest occur.

In the *Pomgnt2*-KO cerebral cortex, Cajal–Retzius cells and subplate neurons ectopically aggregated at meningeal spaces and formed heterotopia (Figs 3 and 4). Given that the emergence of calretinin^+^ectopic clusters corresponded to the timing and region of the basement membrane breach and that fragmented laminin signals were observed over the cluster (Figs 2 and 3), the aberrant aggregation of Cajal–Retzius cells and subplate neurons may be contributing factors in the basement membrane disruption. In normal corticogenesis, Cajal–Retzius cells and subplate neurons are both aligned in two distinct layers beneath the pial surface and form the preplate^24^,^25^,^26^. Several lines of evidence show that the migration and localization of Cajal–Retzius cells in the developing brain are regulated by chemokine signaling, especially CXCL12 (also known as stromal cell-derived factor-1, SDF-1) and its receptor CXCR4^38^,^39^. CXCL12, produced by meningeal cells, enhances the tangential migration of CXCR4-expressing Cajal–Retzius cells and restricts their trajectories to the superficial-most side of the MZ^38^,^39^. Notably, chemokines, including CXCL12, are highly basic proteins and bind to negatively charged glycans, such as glycosaminoglycans^40^,^41^, and this chemokine–carbohydrate interaction is known to regulate the local distribution of chemokines^41^,^42^. Therefore, considering that the ligand-binding heteropolymer on dystroglycan seems to bear negative charges because it contains GlcA, the aberrant glycosylation of dystroglycan located at radial glial end feet facing the meninges may alter the local concentration of CXCL12 around the MZ, resulting in perturbation of the positioning of Cajal–Retzius cells. Although this idea is just a hypothesis and interactions between chemokines and dystroglycan have not yet been examined, it may be a distinct aspect of dystroglycan function.

While the final outcome of the neuronal migration defects in dystroglycanopathy has been identified as the over-migration, behavioral changes in the migrating neurons during their journey to the pial surface are not well characterized. In the *Pomgnt2*-KO cerebral cortex, we revealed that migrating pyramidal neurons display abnormal morphologies in the leading process and Golgi apparatus, indicating a disappearance of polarity and migration directionality (Fig. 5). We also observed preplate splitting failure in the *Pomgnt2*-KO brain (Fig. 4). Preplate splitting is a characteristic process in early neocortical development, whereby the two layers of Cajal–Retzius cells and subplate neurons are divided by pyramidal neurons migrating toward the pial surface, resulting in the generation of the CP^24^. Failure of preplate splitting is a prominent feature of mice with deficiencies in the reelin signaling pathway, such as the *reeler* mutant, Dab1 mutant, and the double mutant of reelin receptors apolipoprotein E receptor 2 and very low-density lipoprotein receptor^43^,^44^,^45^. Neurons lacking reelin signaling cannot maintain their leading processes due to the dysregulation of adhesion molecules, resulting in a defect in somal translocation that leads to preplate splitting failure^28^,^31^,^32^. In addition, neuronal polarity was also affected in *reeler* mice^27^,^46^. These *reeler* neuronal phenotypes apparently resemble the behaviors of migrating neurons in the *Pomgnt2*-KO brain (Fig. 5). Thus, we examined reelin signaling in the *Pomgnt2*-KO brain, but found that it was unchanged (Fig. 7). Previous studies showed that the reelin signaling triggered the recruitment of N-cadherin to the neuronal cell surface and the activation of integrin α5β1, enabling neurons to attach to scaffolds present in the MZ, such as the Cajal–Retzius cell membrane and fibronectin^28^,^31^,^32^. Since reelin signaling is intact in the *Pomgnt2*-KO brain, we speculate that neurons may be unable to maintain their leading processes and rise due to the loss of those anchorage substances caused by the disappearance of the basement membrane and/or ectopic cluster formation. Therefore, this study highlights the essential role of glycans on dystroglycan in the maintenance of the neocortical environment required for the neuronal positioning. Although POMGNT2 seems to be expressed by both radial glial cells and migrating neurons because its mRNA is found throughout the developing mouse brain including the ventricular zone and CP^47^, dystroglycan shows a restricted expression in radial glial cells and is not likely to function in neurons during the development of the cortical lamination^21^,^23^. These observations indicate that, together with dystroglycan, POMGNT2 functions in radial glial cells but not in migrating neurons during the brain development^21^,^23^. Our findings indicate the importance of the appropriate environment in the cerebral cortex in addition to the intrinsic machinery in the neuron for proper migration and layer formation of pyramidal neurons.

## Methods

### Antibodies

The following primary antibodies were used in this study: IIH6 monoclonal antibody (mAb) (Millipore, Billerica, MA), anti-laminin polyclonal antibody (pAb) (Sigma-Aldrich, St. Louis, MO), anti-CD31 mAb (BD Biosciences, San Jose, CA), anti-calretinin pAb (Millipore), anti-MAP2 mAb (Millipore), anti-reelin mAb (Chemicon, Temecula, CA), anti-reelin pAb (R&D Systems, Minneapolis, MN), anti-Pax6 pAb (Covance, Emeryville, CA), anti-GFP pAb (Clontech, Palo Alto, CA), anti-GM130 mAb (BD Biosciences), anti-Nestin mAb (Cell Signaling Technology, Danvers, MA), anti-actin pAb (Millipore), anti-Dab1 pAb (Millipore), anti-phosphotyrosine mAb (clone 4G10: Millipore), anti-brn1 pAb (Santa Cruz Biotechnology, Dallas, TX), and anti-ctip2 mAb (abcam, Cambridge, UK). As secondary antibodies, the horseradish peroxidase (HRP)-conjugated anti-mouse IgG and anti-rabbit IgG (Invitrogen, Carlsbad, CA) were used for immunoblotting, and Alexa Fluor 488- or 546-conjugated anti-mouse IgG, anti-rabbit IgG, and anti-rat IgG (Invitrogen) were used for immunofluorescence.

### Mice

The *Pomgnt2*-KO mouse was previously established^18^. All animal experiments were conducted according to the Fundamental Guidelines for Proper Conduct of Animal Experiments and Related Activities in Academic Research Institutions under the jurisdiction of the Ministry of Education, Culture, Sports, Science and Technology of Japan and approved by the Committees for Animal Experiments of Kyoto University and Nagoya City University. For timed pregnancy mating, noon on the plug date was considered as E0.5.

### Immunohistochemistry

Embryos or dissected brains were fixed in phosphate-buffered saline (PBS) containing 4% paraformaldehyde, cryoprotected in PBS containing 30% sucrose, and embedded in OCT compound (Sakura Finetek, Tokyo, Japan). Coronal sections (12 or 20 μm thick) were prepared from the embedded tissues and incubated for 20 min at room temperature in blocking solution (PBS containing 3% bovine serum albumin and 0.1% Triton X-100). Sections were then incubated with primary antibodies overnight at 4 °C followed by incubation with secondary antibodies at room temperature for 2 h. 4’,6-Diamidino-2-phenylindole (DAPI) was used for nuclear counterstaining. For the morphological analysis of the Golgi apparatus, Golgi morphologies visualized by GM130 staining were split into three categories: radial, not radial, and compact. Among the thin and extended Golgi bodies, those with a vertical orientation (angle range ± 30° from the vertical line to the pial surface) were assigned into the “radial” category, and those with other orientations were considered “not radial”. Golgi bodies with globular and compact shapes were assigned to the “compact” category.

### Expression plasmid

An expression vector for Venus (a GFP variant with the enhanced fluorescence) attached to a membrane-targeted palmitoylation signal of GAP-43 (pCAGGS-GAP-Venus)^48^ was kindly provided by Drs. Y. Yoshihara and A. Miyawaki (RIKEN Brain Science Institute, Wako, Saitama, Japan).

### *In utero* electroporation

*In utero* electroporation was conducted according to an established procedure^49^. Briefly, timed-pregnant mice were anesthetized and the uterine horns were exposed. The plasmid solution containing 1 μg/μl of cDNA and 0.01% Fast Green solution was injected into the lateral ventricle of the embryonic brain at E12.5 using a pulled glass capillary. The head was clasped by a pair electrode (NEPA Gene, Chiba, Japan) and electric pulses (30 V for 50 ms, five times in 950-ms intervals) were delivered using an electroporator (NEPA Gene).

### Preparation of mouse brain lysates

Whole brains of E16.5 control and *Pomgnt2*-KO mice were homogenized using a polytron homogenizer in nine volumes of homogenization buffer [20 mM Tris–HCl (pH 7.4), containing 150 mM NaCl, 1 mM ethylenediaminetetraacetic acid (EDTA), 1 mM sodium orthovanadate, and a protease inhibitor cocktail (Nacalai Tesque, Kyoto, Japan)]. The homogenate was centrifuged at 1,000 × *g* for 10 min at 4 °C to remove nuclei and large debris. Then, Triton X-100 (1% final concentration) was added to the supernatant, followed by incubation for 30 min at 4 °C and centrifugation at 105,000 × *g* for 60 min at 4 °C. The resulting supernatant was used as the brain lysate for biochemical experiments.

### Immunoprecipitation and immunoblotting

For immunoprecipitation, the brain lysate was incubated with the primary antibody and protein G-conjugated Sepharose (GE Healthcare, Little Chalfont, Buckinghamshire, UK) for 2 h at 4 °C. The beads were recovered by centrifugation (400 × *g* for 2 min) and washed three times with Tris-buffered saline containing 0.1% Triton X-100. The beads were boiled in Laemmli sample buffer, and eluted proteins were subjected to sodium dodecyl sulfate–polyacrylamide gel electrophoresis (SDS-PAGE). For immunoblotting, solubilized proteins were separated by SDS-PAGE on a 10% polyacrylamide gel and transferred onto nitrocellulose membranes. After blocking with 5% nonfat dry milk in PBS containing 0.05% Tween 20, membranes were incubated with primary antibodies, followed by the incubation with HRP-conjugated secondary antibodies. Protein bands were detected using the Super Signal West Pico chemiluminescence reagent (Thermo Fisher Scientific, Waltham, MA) and a LAS-3000 Luminoimage Analyzer (Fujifilm, Tokyo, Japan).

### Statistics

Statistical significance was determined by a two-tailed Student’s *t*-test. *P*-values of <0.05 were considered statistically significant.

## Additional Information

**How to cite this article**: Nakagawa, N. *et al.* Ectopic clustering of Cajal-Retzius and subplate cells is an initial pathological feature in *Pomgnt2*-knockout mice, a model of dystroglycanopathy. *Sci. Rep.* **5**, 11163; doi: 10.1038/srep11163 (2015).

## Supplementary Material

Supplementary Information
