# Supplementary material for: Ectopic clustering of Cajal–Retzius and subplate cells is an initial pathological feature in Pomgnt2-knockout mice, a model of dystroglycanopathy
Source: Sci Rep. 2015 Jun 10;5:11163. doi: 10.1038/srep11163 (PMC4461912; doi:10.1038/srep11163)
Supplement: Supplementary Information [file srep11163-s1.pdf]

Supplementary Information

Ectopic clustering of Cajal–Retzius and subplate cells is an initial pathological feature in *Pomgnt2*-knockout mice, a model of dystroglycanopathy

Naoki Nakagawa[1], Hirokazu Yagi[2], Koichi Kato[2,3], Hiromu Takematsu[1], and Shogo Oka[1]

[1]Department of Biological Chemistry, Human Health Sciences, Graduate School of Medicine, Kyoto University, 53 Kawahara-cho, Shogoin, Sakyo-ku, Kyoto 606-8507, Japan; [2]Graduate School of Pharmaceutical Sciences, Nagoya City University, 3-1 Tanabe-dori, Mizuho-ku, Nagoya 467-8603, Japan; [3]Okazaki Institute for Integrative Bioscience and Institute for Molecular Science, National Institutes of Natural Sciences, 5-1 Higashiyama Myodaiji, Okazaki 444-8787, Japan.

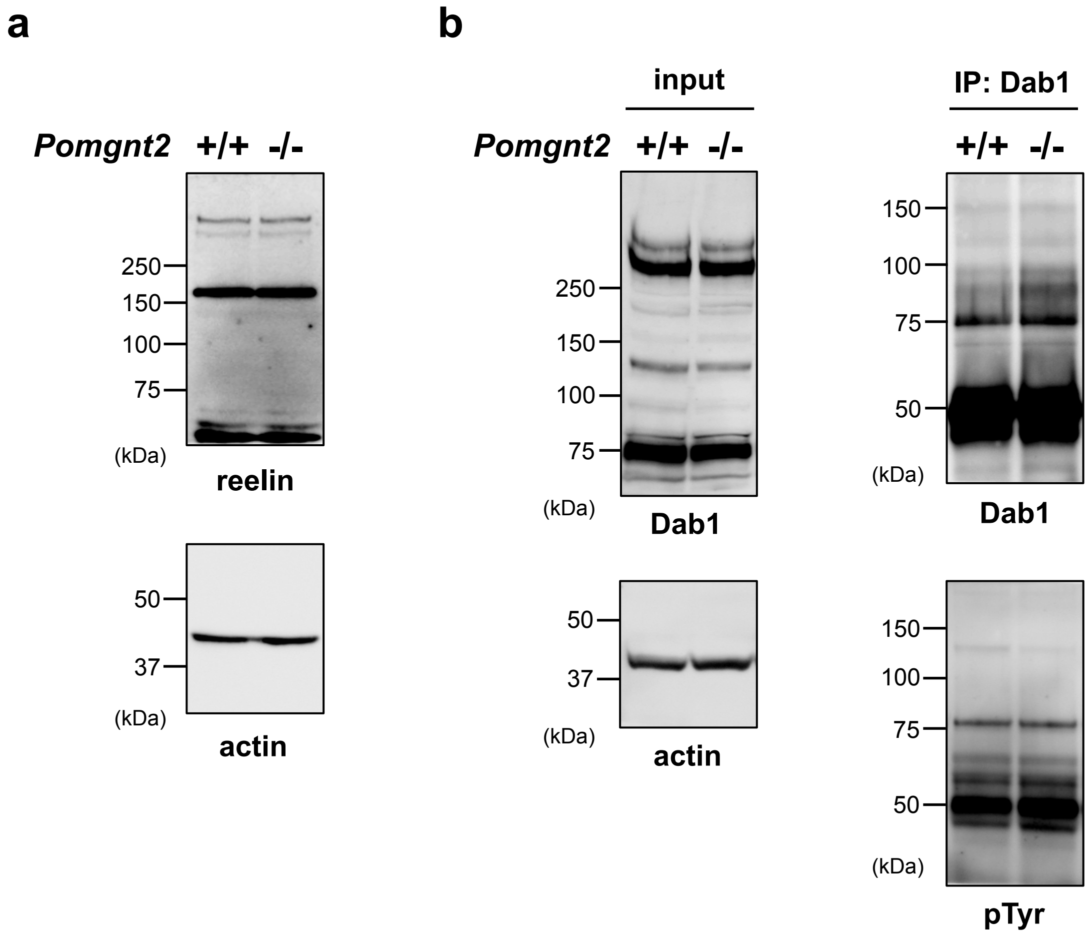

Fig. S1 | Full-length blots.
The regions of interest are highlighted in main Figs: (a) Fig. 7a; (b) Fig. 7b.
